# Novel mitophagy inducer TJ0113 alleviates pulmonary inflammation during acute lung injury

**DOI:** 10.3389/fphar.2025.1590458

**Published:** 2025-07-09

**Authors:** Zhengyuan Liu, Danruo Fang, Kaijun Chen, Lingling Dong, Huaqiong Huang, Zhihua Chen

**Affiliations:** Key Laboratory of Respiratory Disease of Zhejiang Province, Department of Respiratory and Critical Care Medicine, Second Affiliated Hospital of Zhejiang University School of Medicine, Hangzhou, Zhejiang, China

**Keywords:** acute lung injury, mitophagy inducer, TJ0113, NF-κB, NLRP3 inflammasome

## Abstract

**Introduction:**

Acute lung injury (ALI) is a severe respiratory disease with limited effective therapeutic options. Recent studies have highlighted mitochondrial damage as a crucial factor in the progression of ALI. Mitophagy, which facilitates the removal of damaged mitochondria, has been shown to reduce inflammation. Our collaborators constructed a small molecule mitophagy inducer, TJ0113. TJ0113 has been approved to initiate a Phase I clinical trial for Alport syndrome and a Phase II trial for Parkinson's disease in China. Therefore, we explored the potential of TJ0113 as a novel therapeutic for ALI.

**Methods:**

The mitophagy-inducing potential of TJ0113 was assessed in HEK293T cells. The anti-inflammatory effects of TJ0113 were further evaluated in vivo using a mouse model of lipopolysaccharide (LPS)-induced ALI and in vitro using LPS-stimulated bone-marrow-derived macrophages (BMDMs).

**Results:**

TJ0113 selectively induced mitophagy in damaged mitochondria. Furthermore, the PINK1–Parkin pathway was identified as a specific mitophagy pathway induced by TJ0113. In LPS-induced ALI mouse model, intraperitoneal injection of TJ0113 significantly reduced lung inflammation and mortality. *In vitro*, TJ0113 significantly inhibited the expression of LPS-induced inflammatory cytokines in BMDMs. Finally, we found that TJ0113 inhibited LPS-induced inflammation by inducing mitophagy and inhibiting nuclear factor κB (NF-κB) and inflammasome activation.

**Conclusion:**

TJ0113 alleviates LPS-induced inflammation by inducing mitophagy and inhibiting NF-κB and inflammasome activation. Its selective action on damaged mitochondria suggests minimal side effects, positioning TJ0113 as a promising therapeutic candidate for ALI.

## 1 Introduction

Acute lung injury (ALI) and acute respiratory distress syndrome (ARDS) are prevalent and severe respiratory diseases (Matthay et al., 2019). ARDS and ALI are continuous pathological processes, and ARDS represents the late manifestation of ALI (Thompson et al., 2017). The incidence rates of ALI and ARDS are 70/100,000 and 59/100,000, respectively, with a fatality rate of up to 40% (Meyer et al., 2021). Furthermore, the global COVID-19 pandemic has significantly increased the incidence and mortality of ALI/ARDS (Beitler et al., 2022; Fan et al., 2020). ALI progresses rapidly, and currently, no specific pharmacological treatments have demonstrated clinical efficacy (Fan et al., 2018; Yadav et al., 2017). The pathogenesis of ALI/ARDS is highly complex, and the key molecular mechanisms remain incompletely understood. Therefore, further elucidating the pathogenesis of ALI/ARDS and developing targeted therapies for their prevention and treatment are crucial.

Mitochondrial dysfunction is recognized as a critical mediator in the pathogenesis of ALI (Jiang et al., 2024; Long et al., 2022). Mitochondrial damage leads to overproduction of reactive oxygen species (ROS) and release of mitochondrial DNA (mtDNA) into the cytoplasm, which act as damage-associated molecular patterns (DAMPs) to trigger innate immune responses (Jiang et al., 2024). These mitochondrial DAMPs are recognized by two major inflammatory pathways: the inflammasome and the nuclear factor kappa B (NF-κB) signaling pathway, both of which play critical roles in the pathogenesis of ALI. The NACHT, LRR and PYD domains-containing protein 3 (NLRP3) inflammasome is a representative inflammasome. NLRP3 responds to ROS and mtDNA by recruiting and activating caspase-1, which in turn cleaves pro-IL-1β and pro-IL-18 into their mature inflammatory forms (Nakahira et al., 2011; Zhou et al., 2011). Concurrently, damaged mitochondria activate NF-κB signaling via recruitment of NEMO and phosphorylation of IKKα/β, leading to the nuclear translocation of NF-κB and transcriptional upregulation of numerous proinflammatory cytokines such as tumor necrosis factor alpha (TNF-α), interleukin-6 (IL-6), chemokine (C-X-C motif) ligand 1 (CXCL1) and CXCL2 (Harding et al., 2023). Persistent activation of these pathways exacerbates lung injury, making them attractive therapeutic targets.

Mitophagy, a conserved form of selective autophagy, plays a crucial role in maintaining mitochondrial quality by eliminating damaged or depolarized mitochondria (Picca et al., 2023). During mitophagy, dysfunctional mitochondria are sequestered into autophagosomes and subsequently degraded in autolysosomes (Palikaras et al., 2015). By clearing damaged mitochondria, mitophagy effectively reduces the accumulation of ROS and mtDNA. Consequently, mitophagy serves as a negative regulator of inflammation by suppressing the activation of NF-κB signaling and the NLRP3 inflammasome (Liu et al., 2022; Kim M. J. et al., 2016). Several studies have shown that enhancing mitophagy via molecules such as PPAR-gamma coactivator α, Sestrin2, heme oxygenase-1 (HO-1), and RUNX1 can protect against oxidative lung damage and mitigate ALI progression (Wu et al., 2021; Kim M. J. et al., 2016; Liu et al., 2021; Shi et al., 2019; Tang et al., 2023). However, despite this promising potential, no clinically available drugs currently target mitophagy for the treatment of ALI.

Recently, our collaborators identified UMI-77, a BH3 mimetic that promotes mitophagy by disrupting the myeloid leukemia 1 (MCL-1) and Bax/Bak interaction, enabling MCL-1 to bind with microtubule-associated protein light chain 3 (LC3) and trigger mitophagy (Cen et al., 2020). However, UMI-77 lacks specificity and induces non-selective autophagy, affecting not only damaged but also normal mitochondria, raising concerns about off-target effects. To address this limitation, a novel small molecule TJ0113 was developed, characterized by a naphthylamine core structure (Xia Hongguang et al., 2024). TJ0113 is designed to selectively trigger autophagy in damaged mitochondria while sparing functional ones, offering a more targeted strategy for mitochondrial quality control.

In this study, we explored the therapeutic potential and mechanism of TJ0113 in lipopolysaccharide (LPS)-induced ALI. Specifically, we examined whether TJ0113 could enhance mitophagy and attenuate inflammation by inhibiting NF-κB and NLRP3 inflammasome activation. Using both *in vitro* and *in vivo* models, we evaluated its ability to modulate mitochondrial homeostasis and reduce lung injury. Given the rapid onset and high mortality of ALI, and the current lack of effective pharmacologic interventions beyond supportive care, our findings may offer a novel mitophagy-targeted therapeutic approach for ALI.

## 2 Materials and methods

### 2.1 HEK293T cell culture and treatment

HEK293T cells were purchased from the American Type Culture Collection cell bank and cultured in RPMI 1640 (Gibco, C11875500BT) supplemented with 10% fetal bovine serum (FBS, Gibco, 10082147) and 1% penicillin-streptomycin under 5% CO_2_ at 37°C. The treatment was performed when the density of the seeding plate reached approximately 90%.

TJ0113 is a gift from the Clinical Pharmacy Research Center of the First Affiliated Hospital of Liangzhu. TJ0113 can be dissolved in 0.1% NaHCO3. Cell viability following TJ0113 treatment was assessed using the CCK-8 Cell proliferation/cytotoxicity assay kit (MedChemExpress, HY-K0301). HEK293T cells (2 × 10^3^ cells/well) were seeded into 96-well plates precultured for 24 h. After treatment with TJ0113, the medium was replaced with fresh medium containing 10 μL of CCK-8 solution, and re-incubated for 1 h. The optical density (OD) at 450 nm was measured using absorbance reader (TECAN, Infinite F50). Experimental groups included: control, TJ0113 at 2.5 μM, 5 μM, and 10 μM for 6, 12, and 24 h.

To model mitochondrial damage *in vitro*, HEK293T cells were treated with 30 μM carbonyl cyanide 3-chlorophenylhydrazone (CCCP, Beyotime, C2003S) as previously described (Kane et al., 2018). For Western blot analysis, HEK293T cells were treated with CCCP and different concentrations or durations of TJ0113. For treated with different concentrations of TJ0113 (6 h), groups were divided as follows: control; TJ0113 (10 μM); CCCP; CCCP + TJ0113 (2.5 μM); CCCP + TJ0113 (5 μM); CCCP + TJ0113 (10 μM). For treated with different concentrations of TJ0113 (10 μM), groups were divided as follows: control; TJ0113 (24 h); CCCP; CCCP + TJ0113 (6 h); CCCP + TJ0113 (12 h); CCCP + TJ0113 (24 h). To mimic ALI, HEK293T cells were treated with LPS (100 ng, 1 μg, 10 μg) for 6 h.

For quantitative PCR (qPCR) analysis, HEK293T cells were co-treated with CCCP (30 μM, 6 h) and TJ0113 (10 μM, 6 h). Groups were divided as follows: DMSO + 0.1% NaHCO_3_, CCCP (30 μM) + 0.1% NaHCO_3_, DMSO + TJ0113 (10 μM), or CCCP (30 μM) + TJ0113 (10 μM).

### 2.2 Detection of mitochondrial membrane potential via JC-1 staining

HEK293T cells were treated with CCCP (30 μM) and TJ0113 (10 μM) for 6 h. Groups were divided as follows: DMSO; CCCP; DMSO + TJ0113. Mitochondrial membrane potential was assessed using the Enhanced Mitochondrial Membrane Potential Detection Kit (Beyotime, C2003S). Cells were resuspended with culture medium and incubated with JC-1 working solution in 37°C for 20 min. Following incubation, cells were centrifuged at 600 *g* for 5 min at 4°C, washed three times with 1× JC-1 staining buffer, and resuspended in the same buffer. Fluorescence was analyzed using flow cytometer (Beckman, CytoFLEX) with excitation at 488 nm and emission at 530 nm, and data were processed using FlowJo software.

### 2.3 Isolation and culture of mouse bone marrow-derived macrophages (BMDMs)

Bone marrow cells were isolated from mice via phosphate buffered saline (PBS) flushing. After centrifugation, red blood cells (RBCs) were removed using RBC lysis buffer (Sigma-Aldrich, R7757). Cells were cultured in RPMI 1640 medium (Gibco, C11875500BT) supplemented with 10% heat-inactivated FBS (Gibco, 10082147), 1% penicillin-streptomycin and 20 ng/mL recombinant mouse macrophage colony-stimulating factor (M-CSF, Novoprotein, CB34) under 5% CO_2_ at 37°C. The medium was replaced on day 3 and further experiments were performed with proliferative nonactivated BMDMs on day 5.

To mimic ALI, BMDMs were treated with LPS or LPS + ATP. For mitophagy inhibition, BMDMs were treated with Midvi-1 (MedChemExpress, HY-15886). For NF-κB signaling pathway detection, BMDMs were treated with LPS (100 ng/mL), TJ0113 (10 μM) and Midvi-1 (10 μM) for 6 h. Experimental groups included: DMSO; TJ0113 + DMSO; Midvi-1; LPS + DMSO; LPS + Midvi-1; LPS + TJ0113; LPS + TJ0113 + Midvi-1.

For inflammasome signaling pathway analysis, BMDMs were treated with LPS (100 ng/mL), TJ0113 (10 μM) and Midvi-1 (10 μM) for 6 h, followed by 0.5 h of 2 μM ATP treatment. Experimental groups included: DMSO; TJ0113+DMSO; Midvi-1; LPS + ATP + DMSO; LPS + ATP + Midvi-1; LPS + ATP + TJ0113; LPS + TJ0113 + Midvi-1.

### 2.4 Animals

Healthy male wild-type C57BL/6 mice (6–8 weeks old, 20–25 g) were purchased from Slack Laboratory (Shanghai Animal Company Limited) and housed in the Laboratory Animal Center of Zhejiang University under specific-pathogen-free conditions. All animals were allowed to acclimate for 1 week before use and had free access to food and water.

### 2.5 ALI mouse model

ALI was induced via intratracheal administration of LPS. Mice were anesthetized with intraperitoneal injection of 1% pentobarbital dissolved in PBS. Using a laryngoscope, 50 μL of PBS or LPS (5 mg/kg for pathological assessments; 20 mg/kg for survival analysis) was administered intratracheally.

For survival analysis, mice received intratracheal LPS (20 mg/kg) and were administered 20 mg/kg TJ0113 or an equal volume of 0.1% NaHCO_3_ via intraperitoneal injection on days 0, 2, 4, 6, and 8. According to the patent, the plasma half-life of TJ0113 in mice is approximately 0.79 h (Xia Hongguang et al., 2024). Mice were divided into two groups: LPS + 0.1% NaHCO_3_ and LPS + TJ0113. The survival percentage of each group were monitored daily for 10 days.

For pathological assessments, mice received intratracheal LPS (5 mg/kg) and were administered 20 mg/kg TJ0113 or an equal volume of 0.1% NaHCO_3_ via intraperitoneal injection 1 h post-LPS administration. Mice were sacrificed 24 h after the final LPS exposure. Groups included: PBS + 0.1% NaHCO_3_; LPS + 0.1% NaHCO_3_; PBS + TJ0113; LPS + TJ0113.

### 2.6 Bronchoalveolar lavage fluid (BALF) collection and analysis

In pathological assessments, mice were sacrificed 24 h post-LPS exposure. The left lung was lavaged three times with 0.4 mL PBS (Solarbio, P1010). Total BALF cells were counted using a cell counter (Count star). BALF was centrifuged at 400 *g* for 10 min at 4°C; supernatants were collected for ELISA and protein assays, and cell pellets were resuspended in PBS and centrifuged on glass slides. Cells stained with Wright–Giemsa stain (Baso, BA-4017). Differential cell counts were performed by counting 200 cells per slide.

### 2.7 Protein concentration determined via BCA assay

Total protein concentration in BALF supernatants was quantified using the Pierce BCA Protein Assay Kit (Thermo Fisher Scientific, 23225). Reagents A and B were mixed at a 50:1 ratio to prepare the working solution. Samples were mixed with the working solution in a 96-well plate, and incubated at 37°C for 30 min in the dark. Absorbance at 570 nm was measured using a microplate reader (TECAN, Infinite F50), and protein concentrations were calculated based on a standard curve.

### 2.8 Lung wet–dry weight ratio

In pathological assessments, mice were sacrificed 24 h after exposure to LPS. The right lung was excised and weighed immediately (wet weight), then dried at 65°C for 36 h and reweighed (dry weight). The wet-to-dry weight ratio was calculated to assess pulmonary edema.

### 2.9 Histological analysis

In pathological assessments, mice left lung tissues were fixed with 4% formaldehyde for 24 h, dehydrated with alcohol, made transparent with xylene, embedded in paraffin, sectioned, and stained with hematoxylin–eosin (HE). The small airway inflammation of similar-size lung tissue sections was observed under a pathological microscope (OLYMPUS, BX61), and the inflammation score was determined according to published references (Kim Y. S. et al., 2016).

### 2.10 Assessment of lung vascular permeability

Pulmonary vascular permeability was evaluated using Evans blue-conjugated albumin extravasation (Rayees et al., 2019; Joshi et al., 2020). Mice were administered Evans blue-conjugated albumin (20 mg/kg) via tail vein injection 30 min before euthanasia. Lung tissues were collected and homogenized. Lung homogenates and plasma samples were incubated with formamide at 55°C for 18 h. The mixtures were then centrifuged to obtain the supernatants. After centrifugation, absorbance of the supernatants was measured at 620 nm using a spectrophotometer (Varioskan Flash, Thermo Fisher Scientific, 5250040). Lung vascular permeability was calculated by transendothelial Evans blue in lungs versus plasma.

### 2.11 Western blot analysis

Whole-cell proteins from HEK293T cells and BMDMs were extracted using 1X SDS-PAGE Sample Loading Buffer (Beyotime, P0015A). Proteins were separated by sodium dodecyl sulfate–polyacrylamide gel electrophoresis (SDS-PAGE) and transferred onto polyvinylidene difluoride (PVDF) membranes (ISEQ00010, Immobilon). Membranes were incubated in blocking buffer for 2 h at room temperature, followed by incubation with primary antibodies overnight at 4°C. The primary antibodies used include: LC3B rabbit polyclonal antibody (Abcam, ab168831), β-actin mouse monoclonal antibody (Cell Signaling Technology, 3700), P-IKK α/β rabbit monoclonal antibody (Cell Signaling Technology, 2697), P-IκBα rabbit monoclonal antibody (Cell Signaling Technology, 2859), p-p65 rabbit monoclonal antibody (Cell Signaling Technology, 3033), p65 rabbit monoclonal antibody (Cell Signaling Technology, 8242), IκBα rabbit polyclonal antibody (Cell Signaling Technology, 9242), p62 rabbit polyclonal antibody (Cell Signaling Technology, 5114), IKKα/β rabbit monoclonal antibody (Cell Signaling Technology, 2697), COXIV rabbit polyclonal antibody (Cell Signaling Technology, 4850), TOM20 mouse monoclonal antibody (Santa Cruz, sc-17764), p-PINK1 rabbit polyclonal antibody (Signalway Antibody, 29297), NLRP3 mouse monoclonal antibody (AdipoGen, AG-20B-0014), Total and Cleaved IL-1β Antibody (Abmart, P50520-1R1S), and caspase-1 rabbit polyclonal antibody (Proteintech, 22915-1-AP). After washing, membranes were incubated with secondary antibodies, including Goat Anti-Rabbit IgG H&L (HRP) (Invitrogen, A32732) and Goat Anti-Mouse IgG H&L (HRP) (Invitrogen, A32723TR). Protein bands were visualized using an enhanced chemiluminescence system (Share-bio, SB-WB011), according to the manufacturer’s instructions. β-actin was used as a loading control. Densitometric analysis was performed using ImageJ software.

### 2.12 RNA isolation and RT-PCR

Total RNA was extracted from lung tissues, HEK293T cells, and BMDMs using TRIzol reagent (Takara Biotechnology, 9109). RNA concentration and purity were assessed using a NanoDrop 2000 spectrophotometer (Thermo Scientific), ensuring A260/A280 ratios between 1.8 and 2.0. Complementary DNA (cDNA) was synthesized from total RNA using reverse transcription reagents (Takara Biotechnology, DRR037A). qPCR was conducted using TB Green^®^ Premix Ex Taq^®^ (Takara) on a StepOnePlus PCR system (Applied Biosystems, Foster City, CA, United States). Relative gene expression levels were analyzed using ABI software and Microsoft Excel, with each sample assessed in triplicate. The real-time RT-PCR-specific primers listed in Table 1 were used to evaluate gene expression.

**TABLE 1:** Real-time RT-PCR-specific primers used to evaluate gene expression.

Primer name	Primer Sequence (5′–3′)
Mouse *β-Actin*	Forward: GGC​TGT​ATT​CCC​CTC​CAT​CG
	Reverse: CCA​GTT​GGT​AAC​AAT​GCC​ATG​T
Mouse *Il-6*	Forward: CCA​AGA​GGT​GAG​TGC​TTC​CC
	Reverse: CTG​TTG​TTC​AGA​CTC​TCT​CCC​T
Mouse *Il-1β*	Forward: GCA​ACT​GTT​CCT​GAA​CTC​AAC​T
	Reverse: ATC​TTT​TGG​GGT​CCG​TCA​ACT
Mouse *Il-18*	Forward: GAC​TCT​TGC​GTC​AAC​TTC​AAG​G
	Reverse: CAG​GCT​GTC​TTT​TGT​CAA​CGA
Mouse *Tnf-α*	Forward: CCC​TCA​CAC​TCA​GAT​CAT​CTT​CT
	Reverse: GCT​ACG​ACG​TGG​GCT​ACA​G
Mouse *Cxcl1*	Forward: CTGGGATTCACCTCAAGAACATC Reverse: CAGGGTCAAGGCAAGCCTC
Mouse *Cxcl2*	Forward: CCA​ACC​ACC​AGG​CTA​CAG​G
	Reverse: GCG​TCA​CAC​TCA​AGC​TCT​G
Human *PINK1*	Forward: GCC​TCA​TCG​AGG​AAA​AAC​AGG
	Reverse: GTC​TCG​TGT​CCA​ACG​GGT​C
Human *PARK2*	Forward: GTG​TTT​GTC​AGG​TTC​AAC​TCC​A
	Reverse: GAA​AAT​CAC​ACG​CAA​CTG​GTC
Human *β-Actin*	Forward: CAT​GTA​CGT​TGC​TAT​CCA​GGC
	Reverse: CTC​CTT​AAT​GTC​ACG​CAC​GAT
Human *BNIP3*	Forward: CAG​GGC​TCC​TGG​GTA​GAA​CT
	Reverse: CTA​CTC​CGT​CCA​GAC​TCA​TGC
Human *FUNDC1*	Forward: CCT​CCC​CAA​GAC​TAT​GAA​AGT​GA
	Reverse: AAA​CAC​TCG​ATT​CCA​CCA​CTG

### 2.13 ELISA

The BALF was collected from mice in pathological assessments experiment, in which mice were divided into four groups: PBS + 0.1% NaHCO3; LPS + 0.1% NaHCO3; PBS + TJ0113; LPS + TJ0113. Cell culture supernatants were obtained from BMDMs. All samples were centrifuged at 400 *g* for 10 min at 4°C, and supernatants were stored at −80°C until analysis. ELISA kits used included mouse chemokine (C-X-C motif) ligand 1 (CXCL1, R&D systems, MKC00B), mouse CXCL2 (R&D systems, MM200), mouse interleukin-6 (IL-6) (R&D systems, M6000B), mouse IL-18 (Neobioscience, EMC011.96), mouse IL-1β (DAKEWE, 1210122), and mouse tumor necrosis factor alpha (TNF-α, DAKEWE, 1217202).

### 2.14 Statistical analysis

GraphPad Prism 8.0 software was used for data processing and plotting. All results are presented as arithmetic mean ± SEM. Differences between two groups were identified using a t-test and between multiple groups using one-way ANOVA, or two-way ANOVA. A P-value < 0.05 was considered statistically significant.

## 3 Results

### 3.1 TJ0113 selectively induces mitophagy through the PINK1–Parkin pathway

**FIGURE 1:**
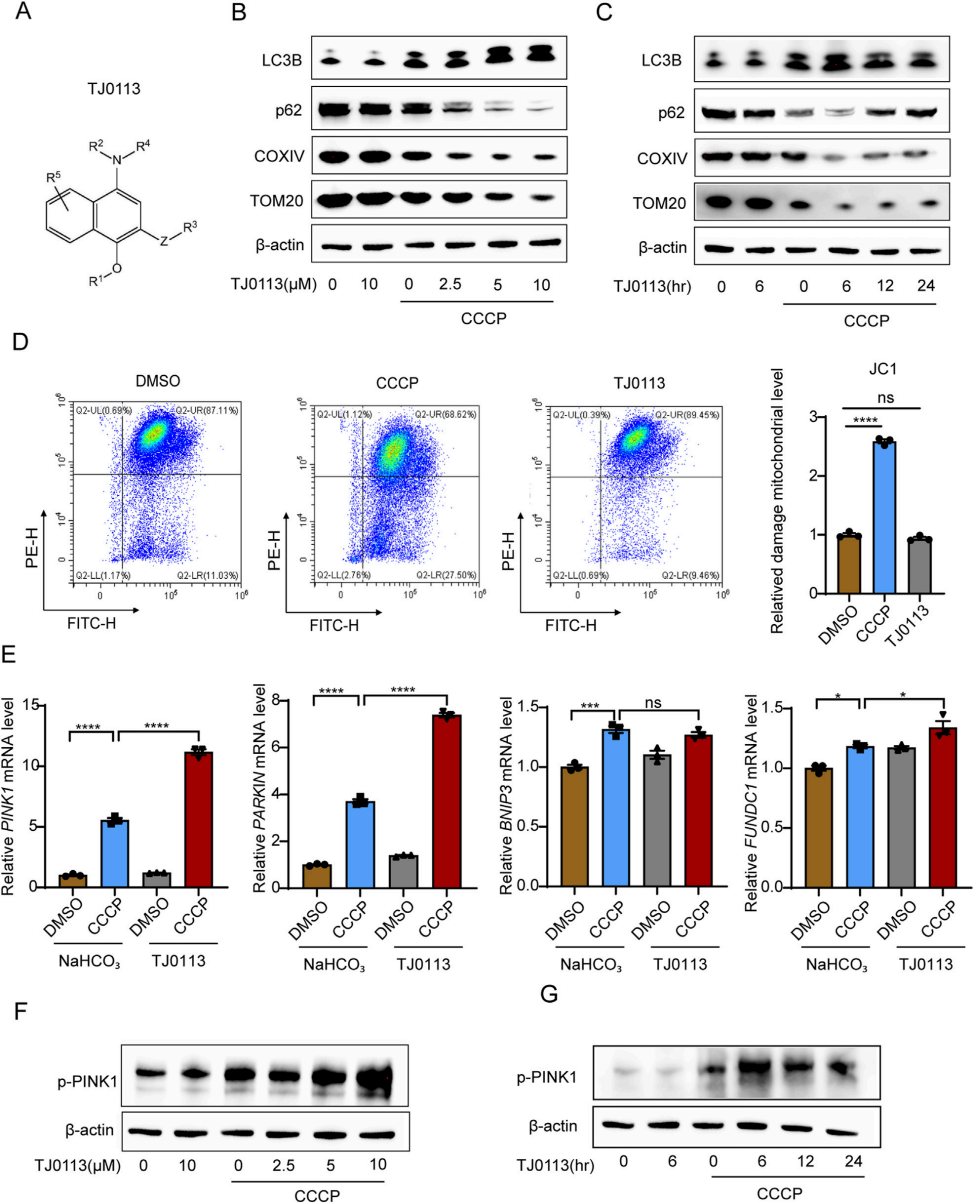
TJ0113 selectively induces mitophagy through PINK1–Parkin pathway. **(A)** Molecular structure of TJ0113. **(B, C)** Western blot analysis of autophagy (LC3B, p62) and mitochondrial proteins (COXIV, TOM20) in HEK293T cells treated with TJ0113 and/or CCCP. **(D)** JC-1 staining shows mitochondrial membrane potential of HEK293T cells. **(E)** Relative mRNA levels of *PINK1*, *PARKIN*, *BNIP3*, and *FUNDC1* in HEK293T cells. **(F, G)** Western blotting of phosphorylated PINK1 (p-PINK1) using samples from panel **(B, C)**. The β-actin was reused due to identical sample sets. β-actin served as loading control. Data: mean ± SEM, n=3. ns, not significant; *P<0.05; **P<0.01; ***P<0.001; ****P<0.0001. **(D, E)** Ordinary one-way ANOVA with Tukey’s multiple comparisons test.

TJ0113 is a small-molecule compound with a naphthylamine structure, designed to induce mitophagy (Figure 1A). To evaluate its function, we first assessed whether TJ0113 induces mitophagy in HEK293T cells. Carbonyl cyanide 3-chlorophenylhydrazone (CCCP), a protonophore that collapses mitochondrial membrane potential, was used as a standard inducer of mitochondrial damage (Kane et al., 2018). Under CCCP-treatment, TJ0113 increased levels of the autophagy marker LC3B while decreasing levels of the autophagic substrate p62, indicative of enhanced autophagic flux (Figures 1B,C; Supplementary Figure S2). A concomitant reduction in mitochondrial proteins—including the outer membrane protein TOM20 and the inner membrane protein cytochrome c oxidase IV (COXIV)—further supported mitophagy induction (Figures 1B,C; Supplementary Figure S2). Importantly, in the absence of CCCP, TJ0113 did not alter TOM20 or COXIV expression (Figures 1B,C; Supplementary Figure S2). JC-1 staining confirmed that TJ0113 did not perturb mitochondrial membrane potential under basal conditions (Figure 1D), and CCK-8 assays demonstrated TJ0113 did not affect cell activity (Supplementary Figure S1). The three canonical pathways that induce mitophagy include receptor-mediated BNIP3-like (NIX, known as BNIP3L as well), FUN14 domain contains 1 (FUNDC1) and the PINK1–Parkin pathway (Sandoval et al., 2008; Liu et al., 2012; Wang et al., 2023). To elucidate the pathway through which TJ0113 induces mitophagy, we first assessed the mRNA expression of key regulators across the three pathways. TJ0113 treatment significantly upregulated PINK1 and Parkin transcripts in the presence of CCCP, while the expression change of BNIP3 and FUNDC1 were not obvious (Figure 1E). Subsequent Western blot analysis confirmed an increase in PINK1 phosphorylation following TJ0113 treatment (Figures 1F,G; Supplementary Figure S2). These findings suggest that TJ0113 mediates mitophagy predominantly via the PINK1-Parkin pathway, independent of receptor-mediated pathways.

### 3.2 TJ0113 alleviates LPS-induced acute lung injury

**FIGURE 2:**
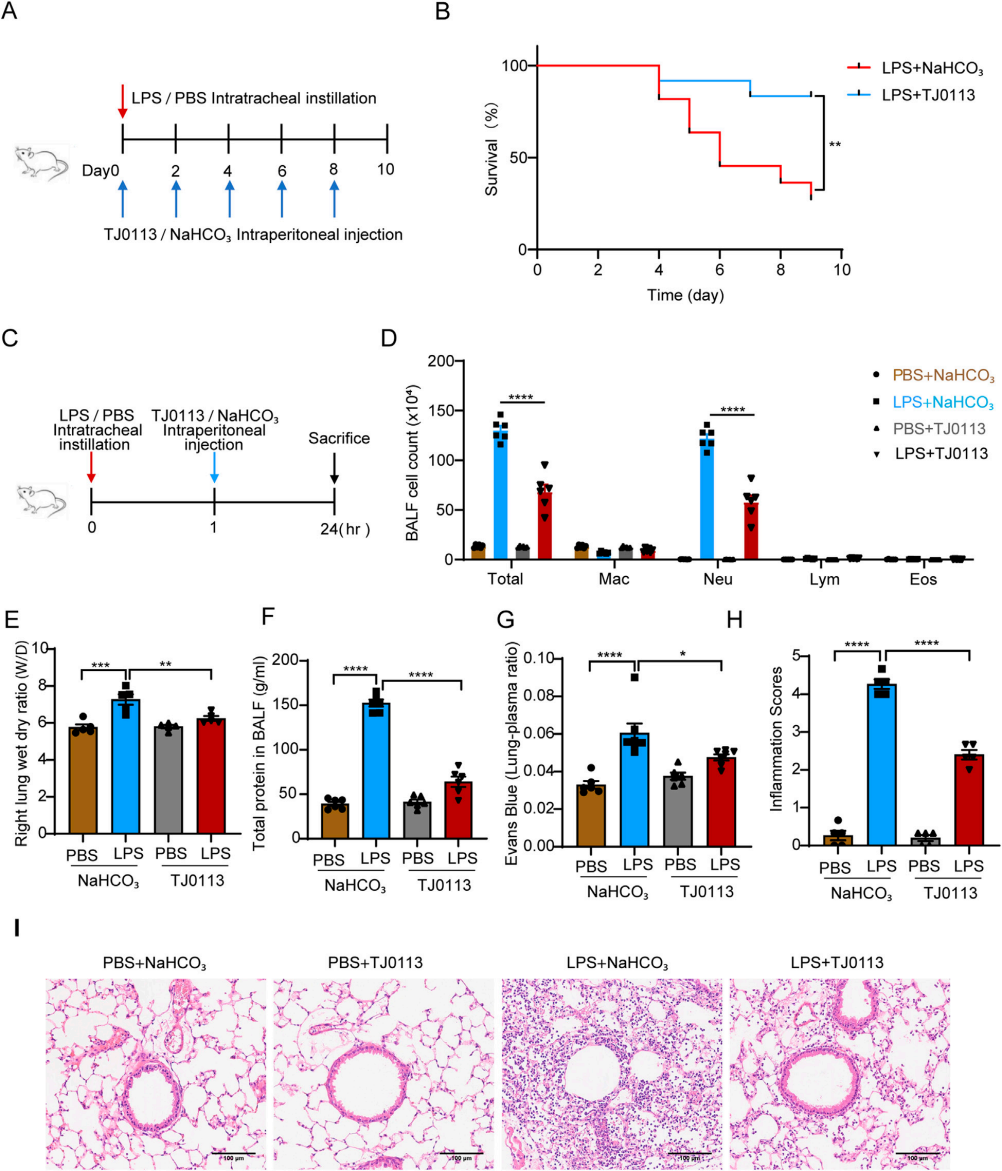
TJ0113 alleviates LPS-induced ALI in a mouse model. **(A)** Schematic of the LPS-induced survival model. **(B)** Survival curves. **(C)** Diagram of LPS-induced ALI model and TJ0113 treatment regimen. **(D)** BALF cell counts: macrophages (Mac), neutrophils (Neu), lymphocytes (Lym), eosinophils (Eos). **(E)** Lung wet/dry (W/D) ratio. **(F)** Total protein content in BALF supernatant. **(G)** Evans blue–albumin lung/plasma ratio. **(H, I)** H&E-stained lung sections and inflammation scores. Scale bar = 100 μm. Data are presented as mean ± SEM. *P<0.05; **P<0.01; ***P<0.001; ****P<0.0001. **(B)** Unpaired t test, **(C)** Ordinary two-way ANOVA with Tukey’s multiple comparisons test, **(E-I)** Ordinary one-way ANOVA with Tukey’s multiple comparisons test.

To investigate the effects of TJ0113 on ALI, we first assessed the impact of LPS on mitochondria and found that LPS indeed induces mitophagy (Supplementary Figure S3), serving as a defensive response to mitochondrial damage. Subsequently, we evaluated the protective effects of TJ0113 on ALI by establishing a mouse survival model (Figure 2A). Mice treated with LPS exhibited significant weight loss, lethargy, and anorexia, whereas TJ0113 administration ameliorated these clinical symptoms. Survival analysis demonstrated a markedly higher survival rate in TJ0113-treated mice compared to the LPS-NaHCO_3_ group (Figure 2B).

We also established an LPS-induced ALI mouse model alongside TJ0113 treatment (Figure 2C). Total cell and neutrophil counts in BALF were significantly reduced in LPS-TJ0113 group compared to the LPS-NaHCO_3_ group (Figure 2D). The lung wet-to-dry weight ratio, indicative of pulmonary edema, was also decreased in the TJ0113 group (Figure 2E). Furthermore, TJ0113 administration led to lower total protein concentrations in BALF supernatants and reduced Evans blue-conjugated albumin leakage (Figures 2F,G), suggesting improved alveolar-capillary barrier integrity. H&E staining further showed reduced infiltration of inflammatory cells around the airway of TJ0113 treated group (Figures 2H,I).

**FIGURE 3:**
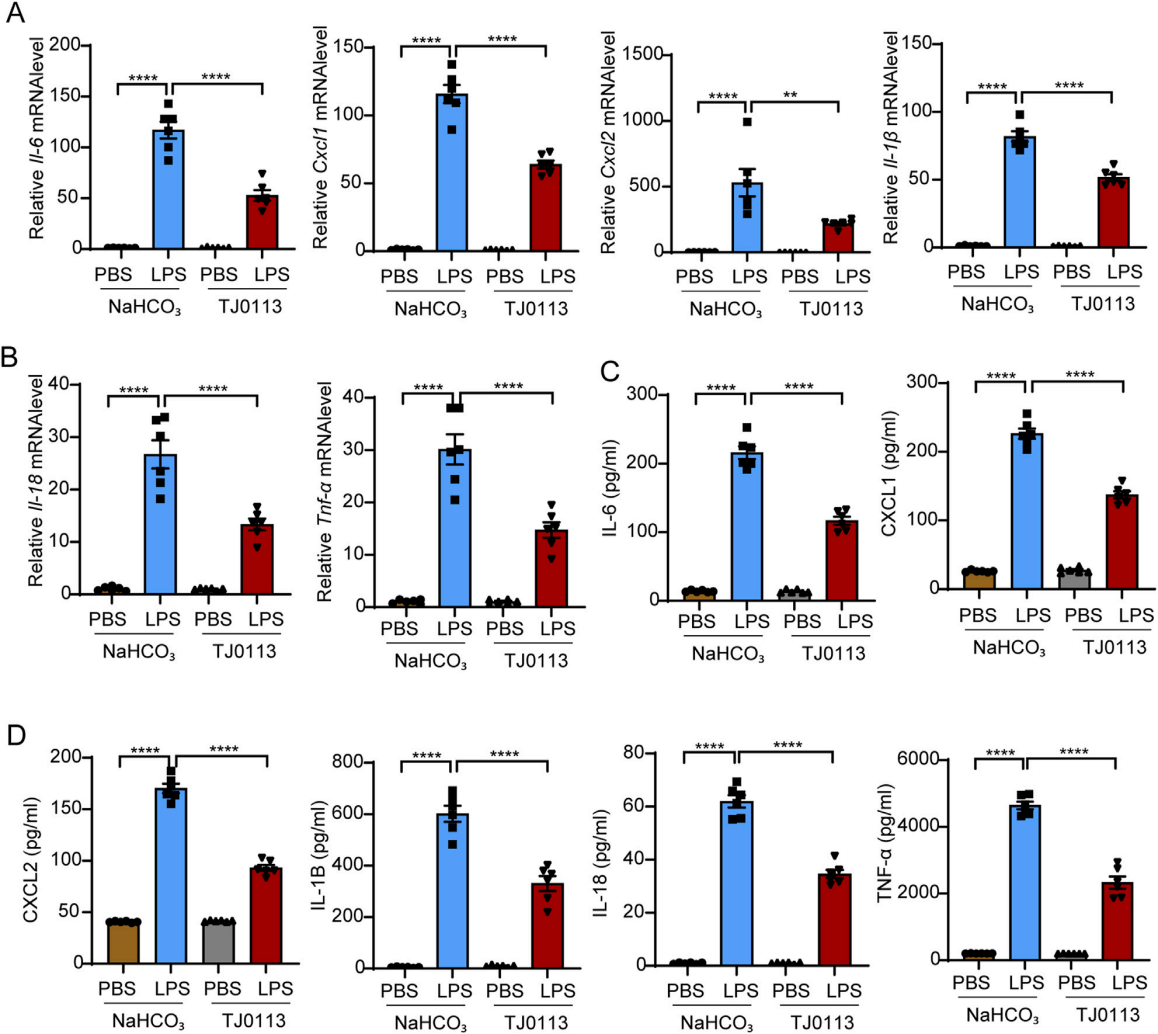
TJ0113 significantly inhibits the cytokine levels in LPS-induced ALI. Mice were divided into four groups as described in Figure 2C: PBS + NaHCO_3_, LPS + NaHCO_3_, PBS + TJ0113, and LPS + TJ0113. **(A, B)** mRNA expression levels of *Il-6*, *Cxcl1*, *Cxcl2*, *Il-1β*, *Il-18*, and *Tnf-α* in lung tissues. **(C, D)** Protein concentrations of IL-6, CXCL1, CXCL2, IL-1β, IL-18, and TNF-α in BALF supernatants were measured by ELISA. Data are presented as mean ± SEM; **, P<0.01; ****, P<0.0001. Ordinary one-way ANOVA with Tukey’s multiple comparisons test.

To further investigate the anti-inflammatory mechanisms of TJ0113, we quantified the expression of pro-inflammatory cytokines. Quantitative PCR analysis of lung tissues showed that TJ0113 significantly downregulated mRNA levels of *Il-6*, *Cxcl1*, *Cxcl2*, *Il-1β*, *Il-18*, and *Tnf-α* (Figures 3A,B). Consistently, ELISA indicated reduced concentrations of these cytokines in BALF supernatants following TJ0113 treatment (Figures 3C,D). Collectively, these findings suggest that TJ0113 mitigates LPS-induced pulmonary inflammation and edema, thereby enhancing survival in a murine ALI model.

### 3.3 TJ0113 attenuates inflammatory response by inhibiting NF-κB activation

**FIGURE 4:**
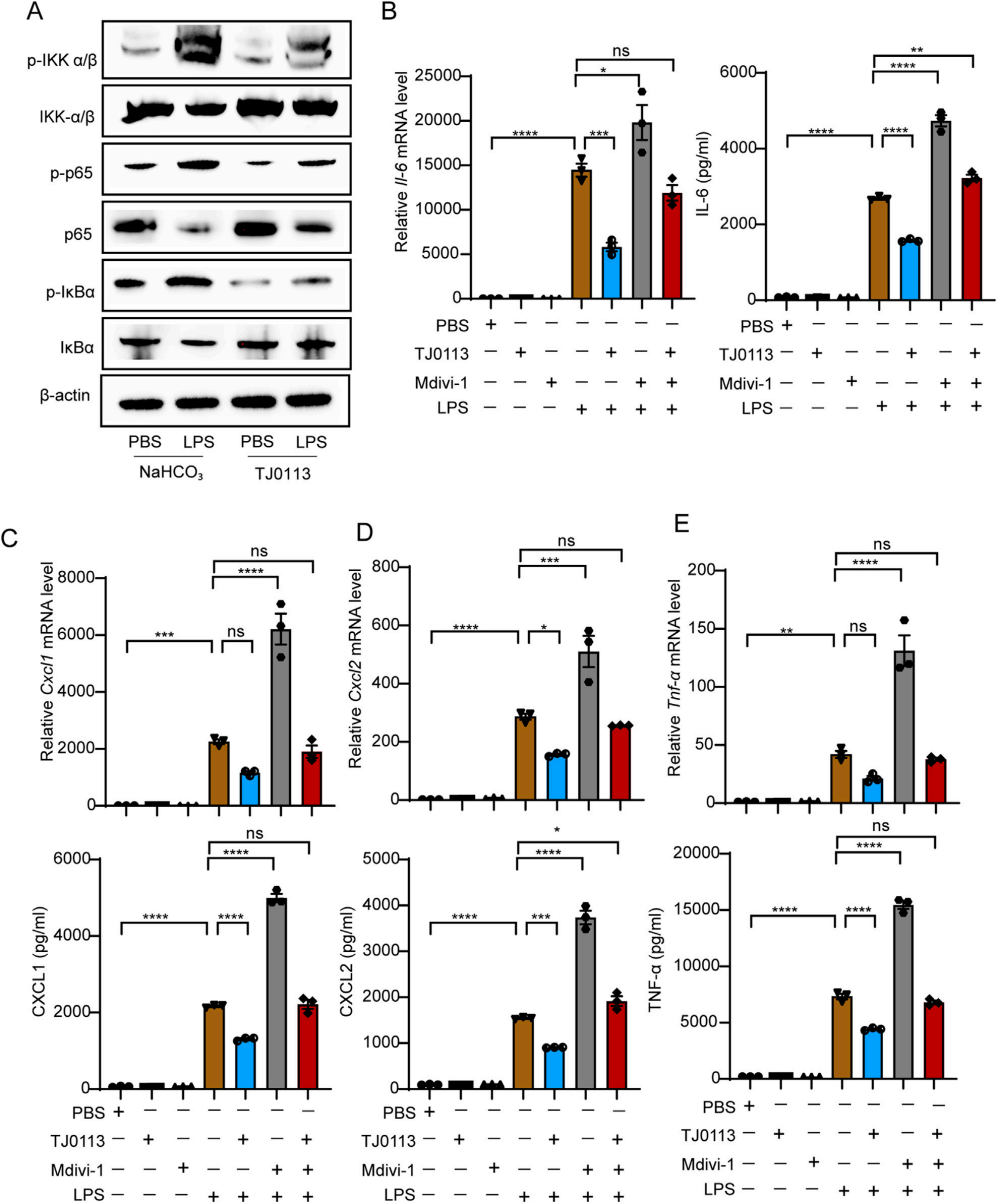
TJ0113 attenuates inflammation by inhibiting NF-κB activation. **(A)** BMDMs were pretreated with TJ0113 (10 μM, 6 h), followed by stimulation with LPS (100 ng/mL, 5 min). Western blotting assessed the expression of p-IKK, IKK-β, IκB, p-IκB, p65, and p-p65. **(B–E)** BMDMs were co-treated with TJ0113 (10 μM) and LPS (100 ng/mL) for 6 h. The mRNA expression level of *Il-6*, *Cxcl1*, *Cxcl2*, and *Tnf-α* and corresponding cytokine concentrations of cell culture supernatant. Data are presented as mean ± SEM, n=3. ns = no significance; **, P<0.01; ***, P<0.001; ****, P<0.0001. Ordinary two-way ANOVA with Tukey’s multiple comparisons test.

Given the critical role of the inflammasome and NF-κB in mitochondrial damage and ALI, we next investigated whether TJ0113 modulates the activation of NF-κB and NLRP3. BMDMs were pretreated with TJ0113 for 6 h, followed by LPS stimulation for 5 min. Western blot analysis showed a reduction in total IKKα/β protein levels and a concomitant increase in phosphorylated IKK α/β (p- IKKα/β) following LPS stimulation. This was accompanied by increased phosphorylation of IκBα (p-IκBα) and decreased total IκBα levels. Phosphorylation of NF-κB p65 subunit (p-p65) also increased, while total p65 levels declined. TJ0113 pretreatment reduced p-IKKα/β, p-IκBα, and p-p65 levels compared to the LPS group (Figure 4A; Supplementary Figure S4).

TJ0113 significantly reduced the mRNA expression of *Il-6*, *Cxcl1*, *Cxcl2*, and *Tnf-α*, which are downstream targets of NF-κB, in LPS-stimulated BMDMs (Figures 4B–E). ELISA results further confirmed reduced levels of these cytokines in the cell culture supernatants (Figures 4B–E). Furthermore, when cells were co-treated with Mdivi-1, a mitophagy inhibitor, the protective effect of TJ0113 was significantly attenuated (Figures 4B–E). These findings indicate that TJ0113 inhibits activation of NF-κB signaling pathway by inducing mitophagy.

### 3.4 TJ0113 attenuates inflammatory response by inhibiting inflammasome activation

**FIGURE 5:**
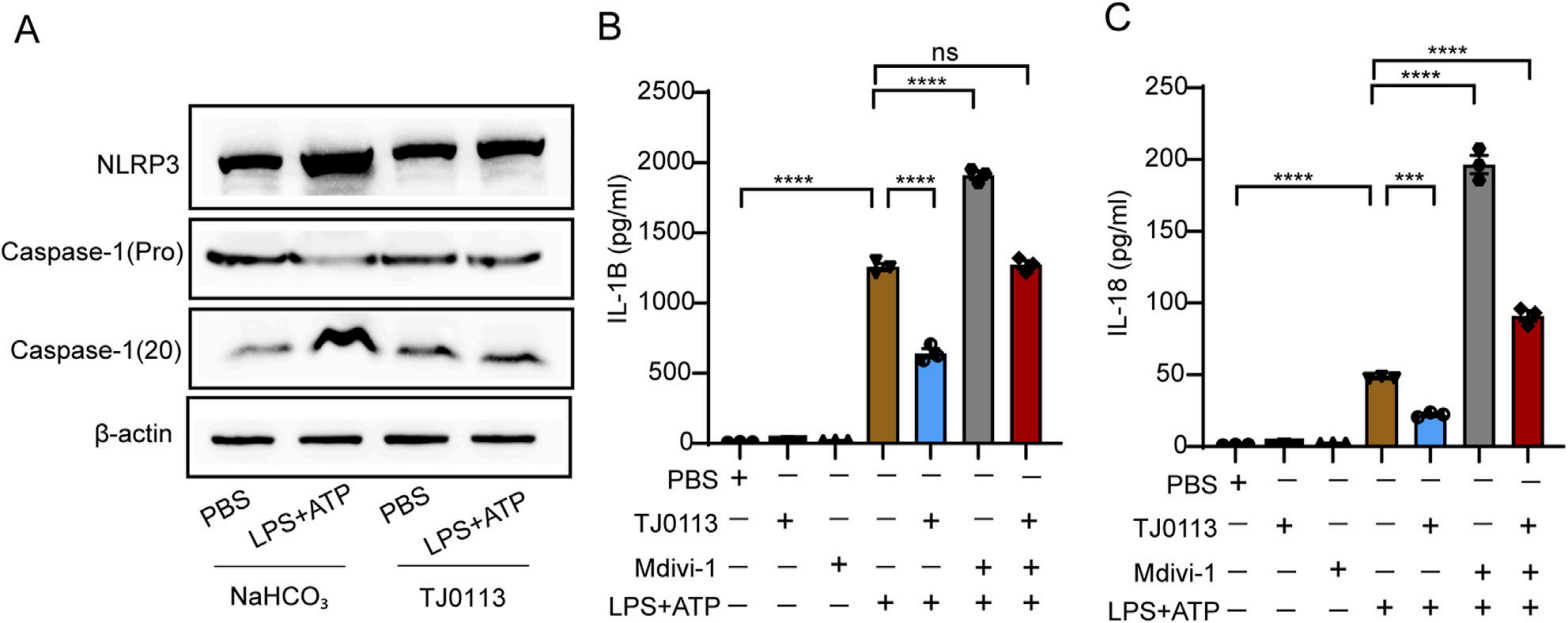
TJ0113 attenuates inflammation by inhibiting inflammasome activation. BMDMs were treated with TJ0113 (10 μM) and LPS (100 ng/mL) for 6 h followed by 30 min ATP (2 μM) treatment. BMDMs were divided into four groups: PBS + NaHCO_3_, LPS-ATP + NaHCO_3_, PBS + TJ0113, and LPS-ATP + TJ0113. **(A)** Western blot analysis assessed the expression of NLRP3, Caspase-1 (pro), Caspase-1 (20). **(B-C)** IL-1β and IL-18 in the culture supernatant were measured by ELISA. Data are presented as mean ± SEM, n=3; ns=no significance; **, P<0.01; ***, P<0.001; ****, P<0.0001. Ordinary two-way ANOVA with Tukey’s multiple comparisons test.

Cleaved caspase-1 (p20) was elevated following LPS plus ATP stimulation, while TJ0113 treatment reduced cleaved caspase-1 (p20) levels (Figure 5A; Supplementary Figure S5). ELISA showed that secretion of mature IL-1β and IL-18 into the supernatant increased upon LPS-ATP stimulation but was significantly reduced by TJ0113 (Figures 5B,C). However, Western blot analysis showed no notable change in intracellular pro-IL-1β protein levels across treatment groups (Supplementary Figures S5D,E). TJ0113 reduces extracellular levels of mature IL-1β and IL-18, while intracellular pro-IL-1β remains unchanged. These results indicated that TJ0113 inhibited caspase-1 mediated cytokine processing. Furthermore, when cells were co-treated with Mdivi-1, the protective effect of TJ0113 was significantly attenuated (Figures 5B,C). Together, these findings demonstrate that TJ0113 suppresses inflammasome activation and cytokine maturation in a mitophagy-dependent manner.

## 4 Discussion

In this study, we explored the therapeutic potential of TJ0113 in LPS-induced ALI mouse model. Our results demonstrate that TJ0113 alleviates pulmonary inflammation and edema by selectively inducing mitophagy via the canonical PINK1–Parkin pathway. Distinct from conventional mitophagy inducers, TJ0113 exhibits mitochondrial specificity, targeting only damaged mitochondria while preserving normal mitochondrial integrity. Mechanistically, TJ0113 suppresses the activation of both NF-κB and NLRP3 inflammasome signaling pathways through mitophagy induction, thereby attenuating the inflammatory response. As a novel and highly selective mitophagy activator, TJ0113 shows considerable promise for clinical translation, particularly in the treatment of ALI and ARDS driven by infectious or inflammatory insults.

Current therapeutic strategies for ALI/ARDS are primarily supportive, relying on interventions such as mechanical ventilation and fluid management. While these approaches may alleviate symptoms, they have not been shown to substantially reduce mortality (Beitler et al., 2022). Although several pharmacological agents—including vasodilators, surfactants, and antioxidants—have demonstrated theoretical benefits and efficacy in preclinical models, none have successfully translated into effective clinical treatments (Creagh-Brown et al., 2009; Perkins et al., 2006; Halliday, 2008; Lee et al., 2013). In contrast to conventional anti-inflammatory drugs that exert broad systemic effects, TJ0113 acts by targeting mitochondrial dysfunction, a pivotal driver of inflammation in ALI. TJ0113 is the first small-molecule mitophagy inducer developed worldwide, holding independent intellectual property rights (Xia Hongguang et al., 2024). It has shown encouraging results across a range of preclinical disease models. Notably, TJ0113 has been approved to initiate clinical trial for Alport syndrome, Parkinson's disease and depression in China (CTR20232426, CTR20243324, CTR20252210). These findings support the potential of TJ0113 as a promising therapeutic candidate for ALI.

Recent studies have delineated three major regulatory pathways of mitophagy: receptor-mediated NIX/BNIP3 and FUNDC1, and the extensively studied PINK1–Parkin pathway (Kodali et al., 2022; Yan et al., 2019). In our investigation of TJ0113-induced mitophagy, we ruled out the involvement of the BNIP3 and FUNDC1 receptor-mediated pathways at the mRNA level, although further validation at the protein level is warranted. Importantly, both mRNA and protein expression analyses confirmed the upregulation of PINK1 and Parkin, supporting the conclusion that TJ0113 primarily induces mitophagy through the canonical PINK1–Parkin signaling pathway.

After confirming that TJ0113 selectively induces mitophagy, we further evaluated its protective effects in both *in vivo* and *in vitro* models of ALI. *In vivo*, TJ0113 significantly attenuated pulmonary inflammation and improved survival rates in ALI mouse model. Alveolar macrophages (AM) are distributed on the surface of the respiratory tract, which constitute the first line of defense against respiratory pathogens (Busse and Kelly, 2003). During ALI, mitochondrial dysfunction is particularly pronounced in macrophages (Xia et al., 2022), and the release of mitochondrial ROS and mtDNA can activate pro-inflammatory signaling cascades, including NF-κB and inflammasome pathways (Liu et al., 2022; Zhang et al., 2015; Zhong et al., 2013; Lv et al., 2017; Yuan et al., 2022; Yang et al., 2020). *In vitro* experiments, LPS-stimulated BMDMs exhibited robust inflammatory responses, which were significantly suppressed by TJ0113, as evidenced by reduced expression and secretion of inflammatory cytokines. However, we currently lack direct evidence that TJ0113 inhibits the accumulation of ROS and mtDNA, or that these mitochondrial DAMPs are the upstream triggers for NF-κB and inflammasome activation in this context. These mechanistic links warrant further investigation.

While the current findings are encouraging, it is important to note that all experiments were conducted in animal models, which may not fully replicate human physiology. Therefore, caution should be exercised when extrapolating these results to clinical settings. Notably, ongoing clinical trials of TJ0113 for other diseases provide a valuable opportunity to further evaluate its safety and pharmacodynamics in humans. Future research should aim to validate these findings in clinical cases of ALI and to elucidate the precise molecular mechanisms underlying the therapeutic effects of TJ0113.

Based on these findings, TJ0113 demonstrates substantial potential by selectively inducing mitophagy, targeting only damaged mitochondria and sparing healthy ones, positioning it as an effective treatment for inflammation. By suppressing NF-κB and inflammasome pathways, thus mitigating LPS-induced inflammation, TJ0113 fills a critical void in existing ALI/ARDS therapies. Clinically, it could enhance outcomes by halting the progression of mitochondrial dysfunction and excessive inflammation, while also reducing side effects. Ongoing research into its mechanisms and potential applications for other inflammatory conditions could solidify TJ0113 as a valuable therapeutic resource.

## Data Availability

The original contributions presented in the study are included in the article/Supplementary Material, further inquiries can be directed to the corresponding authors.
