# Correction: Novel mitophagy inducer TJ0113 alleviates pulmonary inflammation during acute lung injury

**DOI:** 10.3389/fphar.2025.1667879

**Published:** 2025-08-07

**Authors:** Zhengyuan Liu, Danruo Fang, Kaijun Chen, Lingling Dong, Huaqiong Huang, Zhihua Chen

**Affiliations:** Key Laboratory of Respiratory Disease of Zhejiang Province, Department of Respiratory and Critical Care Medicine, Second Affiliated Hospital of Zhejiang University School of Medicine, Hangzhou, Zhejiang, China

**Keywords:** acute lung injury, mitophagy inducer, TJ0113, NF-κB, NLRP3 inflammasome

In the published article, there was an error in the description of the regulatory status of TJ0113. The article incorrectly stated that TJ0113 had received clinical approval for Alport syndrome from both the China National Medical Products Administration and the U.S. Food and Drug Administration. In fact, TJ0113 has only been approved to initiate a Phase I clinical trial in China (CTR20232426) for the treatment of Alport syndrome, and has not received clinical approval for this indication from the U.S. Food and Drug Administration.

A correction has been made to **Abstract**, Paragraph 1. This sentence previously stated:

“TJ0113 has received clinical approval for Alport syndrome from both the China National Medical Products Administration and the U.S. Food and Drug Administration.”

The corrected sentence appears below:

“TJ0113 has been approved to initiate a Phase I clinical trial for Alport syndrome and a Phase II trial for Parkinson’s disease in China.”

A correction has been made to **Discussion**, Paragraph 2. This sentence previously stated:

“Notably, TJ0113 has received clinical approval for the treatment of Alport syndrome from both the China National Medical Products Administration and the U.S. Food and Drug Administration, and is currently undergoing further clinical evaluation for Parkinson’s disease and depression (ClinicalTrials.gov ID: NCT06832540, NCT06596005; CTR20232426).”

The corrected sentence appears below:

“Notably, TJ0113 has been approved to initiate clinical trial for Alport syndrome, Parkinson’s disease and depression in China (CTR20232426, CTR20243324, CTR20252210).”

The original article has been updated.

