# Supplementary material for: Novel mitophagy inducer TJ0113 alleviates pulmonary inflammation during acute lung injury
Source: Front Pharmacol. 2025 Jul 9;16:1590458. doi: 10.3389/fphar.2025.1590458 (PMC12283733; doi:10.3389/fphar.2025.1590458)
Supplement: Supplementary file 1 [file DataSheet1.docx]

Supplementary Material

#
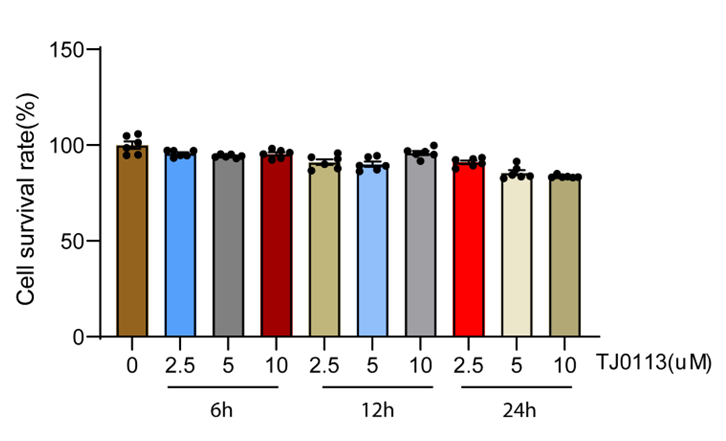
Supplementary Figures

**Supplementary Figure 1.** **The effect of TJ0113 on cell viability was evaluated by CCK8.** HEK293T cells were treated with varying concentrations of TJ0113 (0, 2.5, 5, or 10 μM) for 6, 12, or 24 hours. Cell viability was assessed using the CCK-8 assay. Data are presented as mean ± SEM, n = 6.

**Supplementary Figure 2. Quantification of protein expression in HEK293T cells following TJ0113 and CCCP treatment.** Densitometric analysis of Western blot bands shown in Figure 1. Protein expression levels of LC3B, p62, COXIV, and TOM20 were normalized to β-actin and presented as relative intensity. **(A-D, I)** Quantification corresponding to **Figure 1B and Figure 1F**: HEK293T cells were treated for 6 hours with vehicle control (0.1% NaHCO₃), TJ0113 alone (10 μM), CCCP alone (30 μM), or CCCP (30 μM) combined with varying concentrations of TJ0113 (2.5, 5, or 10 μM). **(E-H, J)** Quantification corresponding to **Figure 1C and Figure 1G**: HEK293T cells were divided into six groups: vehicle control (0.1% NaHCO₃), TJ0113 alone (10 μM, 6 h), CCCP alone (30 μM, 6 h), and CCCP (30 μM, 6 h) co-treated with TJ0113 (10 μM) for 6, 12, or 24 hours. Data are presented as mean ± SEM, n = 3; ns = no significance; *, P < 0.05; **, P < 0.01; ***, P < 0.001; ****, P < 0.0001. Ordinary two-way ANOVA with Tukey's multiple comparisons test.

**Supplementary Figure 3. LPS induces mitophagy in HEK293T cells in a dose-dependent manner.** **(A)** HEK293T cells were treated with increasing concentrations of LPS (0, 100 ng/mL, 1 μg/mL, and 10 μg/mL) for 6 hours. Western blot analysis was performed to detect autophagy markers (LC3B, p62), mitochondrial proteins (COXIV, TOM20), and phosphorylated PINK1 (p-PINK1). β-actin served as the loading control. **(B-F)** Quantitative densitometric analysis of protein bands shown in (A), including LC3B, p62, COXIV, TOM20, and p-PINK1. Quantitative densitometric analysis were normalized to β-actin and presented as relative intensity. Data are presented as mean ± SEM, n = 3; ns = no significant; *, P < 0.05; **, P < 0.01; ***, P < 0.001; ****, P < 0.0001. Ordinary one-way ANOVA with Tukey's multiple comparisons test.

**Supplementary Figure 4. Quantification of NF-κB pathway protein expression in BMDMs following TJ0113 and LPS treatment.** Densitometric analysis of Western blot bands shown in Figure 4A. BMDMs were pretreated with TJ0113 (10 μM) for 6 hours, followed by LPS (100 ng/mL) stimulation for 5 minutes. Cells were divided into four groups: PBS + NaHCO₃, LPS + NaHCO₃, PBS + TJ0113, and LPS + TJ0113. Protein levels of p-IKKα/β, IKKα/β, IκBα, p-IκBα, p65, and p-p65 were quantified. Intensities were normalized to β-actin and presented as relative expression. Data are presented as mean ± SEM, n = 3; ns = not significant; *, P < 0.05; **, P < 0.01; ***, P < 0.001; ****, P < 0.0001. Ordinary one-way ANOVA with Tukey's multiple comparisons test.

**Supplementary Figure 5. Quantification of inflammasome pathway protein expression in BMDMs following TJ0113 and LPS-ATP treatment.** BMDMs were co-incubated with TJ0113 (10 μM) and LPS (100 ng/mL) for 6 hours, followed by ATP (2 μM) for 0.5 hours. Cells were divided into four groups: PBS + NaHCO₃, LPS-ATP + NaHCO₃, PBS + TJ0113, and LPS-ATP + TJ0113. **(A–C)** Densitometric analysis of Western blot bands shown in Figure 5A. **(D, E)** Western blot analysis of pro-IL-1β. Protein levels of NLRP3, pro-Caspase-1, cleaved Caspase-1 (p20) and pro-IL-1β were quantified. Band intensities were normalized to β-actin and presented as relative expression. Data are shown as mean ± SEM, n = 3; ns = not significant; *, P < 0.05; **, P < 0.01; ***, P < 0.001; ****, P < 0.0001. Ordinary one-way ANOVA with Tukey's multiple comparisons test.
